# Malignant Melanoma Misdiagnosis in the Diabetic Foot. What Can We Learn From the Published Literature?

**DOI:** 10.1002/jfa2.70162

**Published:** 2026-05-21

**Authors:** Ivan R. Bristow, Michelle L. Reynolds, Matthew Young

**Affiliations:** ^1^ Health Sciences University Bournemouth UK; ^2^ Private Practice Stockport Manchester UK; ^3^ Diabetes Clinic Edinburgh Royal Infirmary Edinburgh UK

## Abstract

**Background:**

The foot is an independent risk factor for a poorer prognostic outcome in melanoma. Delayed diagnosis may arise because melanoma mimics other benign diagnoses, such as ulceration on the foot, including those with associated with diabetes. Improving outcomes for patients includes raising awareness amongst professionals involved in diabetic foot care. Evaluation of published case studies may help identify common themes to inform practice and improve recognition and referral for lesions requiring biopsy.

**Methods:**

The authors undertook a systematic literature search of PubMed (National Library of Health) (2010–2025) to identify published case studies reporting delayed diagnosis or misdiagnosis of melanoma arising on the skin of the foot or in the toenail in patients with diabetes. Data were collated and tabulated to give basic demographics and assist in identification of common themes. A structured content analysis was undertaken to inform key aspects regarding missed diagnosis.

**Results:**

Following a systematic search of the literature, 1485 papers were identified. After removal of duplicates and irrelevant papers, a total of 86 case studies were identified. A total of 20 cases were reported in patients with diabetes (reported in 19 papers) from 10 countries, including 14 males and 7 females (average age 73 years), with a mean Breslow thickness of 3.58 mm. Melanoma lesions were located on the heels (*n* = 8), forefoot (*n* = 5), toes (*n* = 3), subungual regions (*n* = 2) and in the arch (*n* = 2). The most common misdiagnosis was a diabetic foot ulcer (*n* = 17).

**Conclusion:**

Based on the reviewed cases, distinguishing melanoma from diabetic foot ulceration (DFU) is difficult. Common themes from the content analysis suggest that clues such as ulceration despite lack of the corresponding risk factors, hypergranulation and lack of improvement to be key aspects to suggest an alternative diagnosis. The authors propose a simple acronym, ‘U.L.C.E.R’, as an aide‐mémoire to assist healthcare professionals in the identification of potential melanoma masquerading as DFU.

## 1 Introduction

Cutaneous melanoma is a potentially life‐shortening condition and the fifth most common cancer in the United Kingdom. Melanoma arising on the soles and in the nail unit, the most common primary malignancy in this area [1], has a poorer prognosis for survival [2], possibly due to a range of factors, including anatomy, later recognition, presentation and misdiagnosis [3, 4, 5, 6, 7]. Late diagnosis of melanoma is often due, in part, to its highly variable presentation, which mimics many other benign diagnoses, including diabetic foot ulcers [8]. Improving outcomes for patients with melanoma requires earlier recognition and referral by healthcare professionals.

Podiatrists are uniquely placed to assess foot lesions in patients with diabetes. Previously, the use of the ‘ABCDE’ [9] (Table 1) and ‘CUBED’ acronym [11] (Table 2) have been proposed as means of raising awareness of melanoma and highlighting criteria for earlier referral, but misdiagnoses continue to be reported in the literature.

**TABLE 1:** Summary of the ABCDE acronym for detecting melanoma [10].

A is for asymmetry	One half of the spot is unlike the other half.
B is for border	The spot has an irregular, scalloped, or poorly defined border.
C is for colour	The spot has varying colours from one area to the next, such as shades of tan, brown or black, or areas of white, red, or blue.
D is for diameter	Whilst melanomas are usually greater than 6 mm, or about the size of a pencil eraser, when diagnosed, they can be smaller.
E is for evolving	The spot looks different from the rest or is changing in size, shape, or colour.

**TABLE 2:** The CUBED acronym for detecting melanoma [11].

C	Coloured lesions where any part is not skin colour.
U	Uncertain diagnosis. Any lesion that does not have a definite diagnosis
*B*	*B*leeding lesions on the foot or under the nail, whether the bleeding is direct bleeding or oozing of fluid. This includes chronic “granulation tissue”.
*E*	*E*nlargement or deterioration of a lesion or ulcer despite therapy
*B*	*D*elay in healing of any lesion beyond 2 months.

Analysis of published cases documenting delayed diagnoses or misdiagnoses in patients may highlight common themes valuable in developing further guidance or advice for practitioners responsible for foot care in patients in diabetes.

## 2 Methods

The authors undertook a systematic literature search of PubMed (National Library of Health) [1/1/2010–31/12/2024] to identify any published case studies reporting delayed diagnosis or misdiagnosis of melanoma arising on the skin of the foot or in the toenail in patients. The following search terms used:

1. Foot OR feet OR ‘lower extremity’ OR acral OR plantar OR nail OR leg OR ankle OR sub‐ungual
2. Melanoma
3. Diagn* OR recogn* OR screen*

Papers were limited to those in the English language. Papers were identified and any papers reporting cases of melanoma arising on the feet of patients with diabetes were read in full by two authors (MR and IB). Key data from each case were extracted and tabulated in a spreadsheet to give basic demographic statistics and assist in identification of common themes.

In addition, the discussion sections of all identified papers were copied, and data derived from each case study were subject to a content analysis [10] (a long‐established variant of documentary analysis that enables both qualitative and quantitative measures), enabling a simple numerical measure of the frequency with which concerns were expressed about a possible presence or misdiagnosis of melanoma in nonhealing diabetic foot ulcers.

Frequency counts of either specific key words or themes were identified and recorded, to provide a picture of the features within each discussion section that might imply recognition and consideration of a possible diagnosis of melanoma.

## 3 Results

**FIGURE 1:**
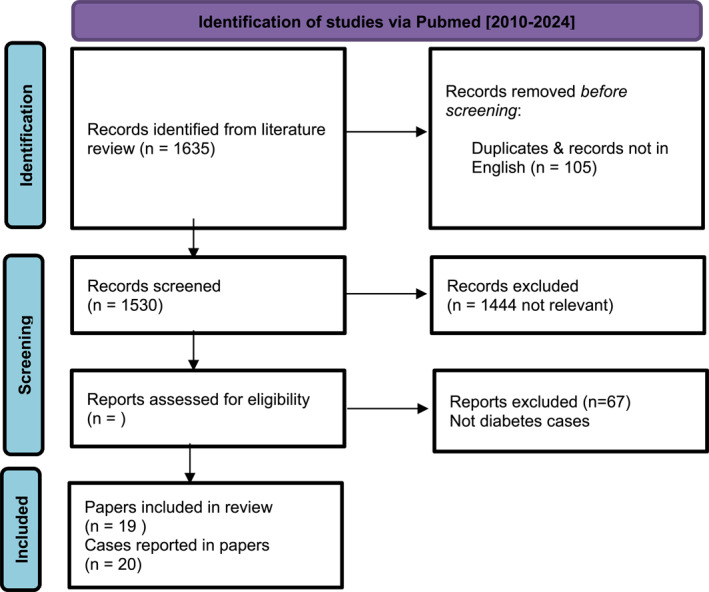
Search strategy.

Following a systematic search of the literature, 1635 papers were identified, of which 1530 written in English. Abstracts were manually reviewed specifically to identify case studies pertaining to report delayed or a missed diagnosis of melanoma. A total of 86 potential case studies were identified (Figure 1). Reading of the full text by the authors uncovered a total of 19 papers reporting 20 cases [12, 13, 14, 15, 16, 17, 18, 19, 20, 21, 22, 23, 24, 25, 26, 27, 28, 29, 30] of misdiagnosed foot melanoma in patients with diabetes (Table 3).

**TABLE 3:** Summary of published case studies.

	Year	Age	Gender	Diabetes type	Left/Right foot	Location	First clinical impression
Gao [12]	2017	78	Female	Type 2	R	Heel (plantar)	Diabetic foot ulcer
Mansur [13]	2016	87	Female	Type 2	L	L5th subungual	Diabetic foot ulcer
Memis [14]	2016	67	Male	Not stated	R	Hallux	Diabetic foot ulcer
Guarneri [15]	2011	86	Male	Type 2	L	Arch (medial)	Diabetic foot ulcer
Han [16]	2018	71	Male	Type 2	R	Toe 4th	Ischaemia
Huang (Pt 1) [17]	2023	70	Female	Type 2	R	Heel	Diabetic foot ulcer
Huang (Pt 2) [17]	2023	66	Female	Type 2	R	R3rd sub‐ungual	Diabetic foot ulcer
Kaneko [18]	2016	80	Male	Type 2	L	Heel (plantar)	Diabetic foot ulcer
Novodorsky [19]	2018	48	Male	Type 2	L	Plantar 1st	Diabetic foot ulcer
Nwabudike [20]	2021	75	Male	Type 2	R	Heel (plantar)	Diabetic foot ulcer
Olivieri [21]	2021	73	Male	Type 2	L	Arch/midfoot plantar	Diabetic foot ulcer
Pagliarello [22]	2012	78	Female	Type 2	?	Toe 4th	Web space infection
Persechino [23]	2016	82	Male	Not stated	?	5th plantar	Diabetic foot ulcer
Shawa [24]	2022	67	Male	Not stated	R	Heel (medial aspect)	Diabetic foot ulcer
Gonzales [25]	2019	68	Male	Not stated	L	Heel	Diabetic foot ulcer
Thomas [26]	2012	81	Male	Not stated	R	Plantar 4th/5th & dorsum	Diabetic foot ulcer
Torres [27]	2010	54	Male	Type 2	R	5th mpj and web space	Diabetic foot ulcer
Wollina [28]	2017	90	Female	Type 2	L	Plantar 2nd/3rd	Slow growing lesion
Yasear [29]	2021	85	Male	Type 2	L	Heel	Diabetic foot ulcer
Zaidi [30]	2016	67	Male	Type 2	R	Lateral border heel	Diabetic foot ulcer

Cases were reported from 10 countries and involved 14 males and 7 females. The average age of patients was 73.6 years (range 48–90 years). Melanomas were located on the heels (*n* = 8), forefoot (*n* = 5), toes (*n* = 3), subungual regions (*n* = 2) and in the arch (*n* = 2). The most common presumed diagnosis was a diabetic foot ulcer (*n* = 17) including three with a presumed ischaemic aetiology. Three others were initially recorded as a web space infection, gangrene and a ‘growing tumour’.

Only three reports noted neuropathy in patients, and 4 cases reported a history of trauma. Histology reported diagnoses of melanoma (*n* = 10), acral lentiginous melanoma (ALM) (*n* = 4), acral melanoma (*n* = 3), amelanotic melanoma (*n* = 2) and nodular melanoma (*n* = 1).

The mean thickness of the reported lesions was 3.58 mm (range 1.5–6.8 mm). At the time of diagnosis, 7 cases were reported as having metastatic spread. Two cases reported recurrence of melanoma following excision. At the conclusion of the case reports, 11 patients were reported to be alive, 2 were deceased and the status of the remaining patients (*n* = 7) was unreported.

### 3.1 Content Analysis

The discussion sections of each paper was analysed for key themes using syntactic (identifying specific keywords or phrases) and thematic analyses (identifying a specific concept or theme). The use of the two analyses meant that both latent and manifest content were assessed. The data were coded by two individuals independently to validate the data captured. Table 4 summarises the key themes uncovered.

**TABLE 4:** Summary of themes arising from the content analysis.

Category code	Key theme	Definition	Frequency	Text exemplar
1	Risk factors absent	Risk factors for ulceration being absent. The reported cases reported that ulceration was occurring in the absence of reasons normally attributable to diabetes.	8	Non‐healing diabetic foot ulcer in the absence of risk factors [30]
2	Longevity	Wounds which fail to heal despite standard care (i.e. good blood sugar level control, off‐loading, antibiosis and redressing) warrant careful attention.	4	Early pathological biopsy should be considered when the wound still does not heal [18]
3	Unusual features	Features within a wound not typical of DFU such as unexplained or excess granulation tissue, spontaneous bleeding, single (or multiple ulcers) appearing in locations not typically associated with ischaemic and neuropathic aetiology and/or with a lack of a history of trauma.	4	Atypical ulcer ‐ such as the presence of granulation tissue [13]
4	Colour	Colour or pigmentation which is irregular or patchy, not consistent with the surrounding skin colour should be consider as potentially suspicious.	2	Irregular patches of brown and black discolouration/pigmentation [20]
5	Enlargement/change	Enlargement despite treatment may be an indication of a malignant lesion.	1	Worsening of the ulcer, failing to respond to treatment [31]

## 4 Discussion

The 19 papers discussed 20 cases of melanoma, predominantly in older patients, which reflects the typical frequency of melanoma, most arise in patients over 60 years of age, which is broadly consistent with other published case series reporting melanoma in this location [31, 32, 33, 34, 35]. The anatomical location of melanoma on the foot has not been widely reported, but the majority of lesions reported in this group were on the heel (40%, *n* = 8), followed by the forefoot (25%, *n* = 5), which is broadly similar to other earlier surveys [31, 36].

The histology within this cohort reported only 2 acral lentiginous melanoma (ALM) and one nodular melanoma—recognised histological sub‐types of melanoma—whereas the remainder were labelled imprecisely as ‘acral’, ‘melanoma’ or ‘amelanotic’—general, nonspecific descriptive terminology for melanoma. Data from previous studies have shown that the ALM subtype accounts for 60% of foot melanomas [37], with superficial spreading and nodular melanoma representing around 40% and 9%, respectively [38]. It is possible many of the misdiagnosed lesions were of the ALM subtype but were not recorded as such.

The mean thickness of lesions was 3.58 mm (range: 1.5–6.8 mm), which is higher than average melanoma thickness at diagnosis [39], with a prognosis under the Breslow thickness scale [40], conferring a 5 years prognosis of around 60%–75%, suggesting that lesions in this series were at a more advanced stage, possibly due to later diagnosis. Previous research studies have shown that typically foot melanomas tend to have a higher Breslow thickness at presentation than comparable lesions on the leg and thigh [6], with delayed diagnosis accounting for the increased morbidity and mortality [4, 41].

The initial presumed diagnosis for most misdiagnosed lesions in this cohort was a diabetic foot ulcer (85% *n* = 17), with 3 of these presumed to have an ischaemic aetiology. The remaining lesions were initially diagnosed as ischaemic necrosis (*n* = 1), a growing tumour (*n* = 1) and a web space infection (*n* = 1). The high rate of initial DFU diagnosis is difficult to explain, but it may be a reflection of the familiarity of the diagnosis over rarer alternatives in a diabetic clinic. Careful assessment of all patients with ulceration of the foot and diabetes is suggested by 8 of the reviewed cases, recognising that, in many cases, the typical causes of diabetic foot ulceration, such as neuropathy and ischaemia, are frequently absent, warranting further detailed assessment to uncover underlying pathology. In addition, none healing and deterioration (despite off loading, antibiosis and monitoring) are discussed in 4 of the cases. A deteriorating or static lesion failing to respond to treatment should warrant further investigation to rule out alternative diagnoses.

Biopsy remains the gold standard for the definitive diagnosis of melanoma and should undertaken without delay when there is suspicion of a potential malignancy in a nonhealing ulcer. Significant delays in recognition and diagnosis have been reported in melanoma affecting the foot [4, 31, 42, 43, 44, 45, 46, 47, 48].

A significant aspect of melanoma arising on the foot is its mimicry of more common, benign lesions, including callus [49], tinea pedis [50, 51, 52], onychomycosis [53, 54], warts [50, 55, 56, 57], hypergranulation tissue [58], bacterial infection [59], ingrowing toenails [60], haematoma [49, 61], arterial disease [62] and benign tumours [63]. Moreover, localised spread by in‐transit metastases mean that melanoma, like other malignancies, may present as multiple lesions on the foot and leg [21].

Foot ulceration, a common complication in diabetes, is also highlighted as a diagnostic pitfall in melanoma misdiagnosis [63, 64, 65, 66, 67, 68, 69, 70, 71, 72]. In one published review of patients with diabetes, 68% of skin malignancies were initially reported as diabetic foot ulcers, with a higher risk of misdiagnosis in older patients [73]. As acral melanoma is more common in older patients [74], this may compound the issue and reinforce the need for a higher degree of suspicion and clinical skill to detect such lesions earlier, particularly in older patients.

However, current published guidance for practitioners in the assessment and management of diabetic foot ulcers, such as the National Institute for Health and Care Excellence [75] and the International Working Group on the Diabetic Foot [76], does not suggest further assessment or biopsy to rule out other causes which may mimic diabetic foot ulceration.

Acronyms are frequently used in medicine because they can increase users' attention, simplify the complexity of information [77] and potentially increase sensitivity, ensuring that diagnoses are missed less frequently [78]. As a result of the categories identified in the content analysis of cases, the authors propose the simple acronym ‘U.L.C.E.R’ (*U*nusual features, *L*ongevity, *C*olour, *E*nlargement and *R*isk factors absent) as an aide‐mémoire to assist healthcare professionals in the identification of potential melanoma masquerading as DFU (summarised in Table 5) and described in detail below.

**TABLE 5:** The proposed “ULCER” acronym.

U	Unusual—spontaneous bleeding or hypergranulation tissue, ulcer located in atypical area for ischaemic or neuropathic lesions.
L	Longevity—a lesion which is static or deteriorating despite treatment.
C	Colour—irregular colour within, or patchy pigmentation around the ulcer
E	Evolution or enlargement despite treatment
R	Risk factors for diabetic ulceration maybe absent such as neuropathy, ischaemia or infection

Unusual features were commonly reported in case studies [12, 15, 17, 30] as potential red flags, such as unexplained or excess granulation tissue, spontaneous bleeding, single (or multiple) ulcers appearing in locations not typically associated with ischaemic and neuropathic aetiology and/or with a lack of a history of trauma.

Longevity was also frequently cited [12, 15, 17, 27]. Wounds that fail to heal despite standard care (good blood sugar level control, off‐loading, antibiosis and redressing) warrant careful attention. Although no papers gave a specific time frame of expected improvement, an earlier guideline for melanoma suggested a 2‐month time frame to consider referral/biopsy when healing is delayed [11].

Colour or pigmentation [12, 19, 29] that is irregular or patchy and not consistent with the surrounding skin colour should be consider as potentially suspicious.

Enlargement [29, 79] despite treatment is an indication of a potentially malignant lesion.

Risk factors for ulceration being absent. The reported cases indicated that ulceration occurred in the absence of factors normally attributable to diabetes, such as infection, trauma, ischaemia and neuropathy [12, 15, 17, 19, 27, 29, 30]. Where no risk factors for ulceration are evident, other causes should considered.

Although melanoma is the focus of this paper, it is acknowledged by the authors that it is not the only skin malignancy reported arising on the skin of the foot of patients with and without diabetes. One 10‐year retrospective study of malignant lesions suggested that melanoma accounted for 80.7% of malignancies arising on the foot with squamous cell carcinoma and basal cell carcinoma accounting for 10.7% and 4.1%, respectively [80]. It is suggested that the proposed acronym may have utility for these rarer lesions, which also frequently exhibit similar characteristics, such as longevity, evolution and unusual features.

## 5 Conclusion

Melanoma arising on the foot is unusual but has a poor prognosis particularly when compared with cutaneous melanoma elsewhere. A systematic review of literature has demonstrated that melanoma arising on the foot in patients with diabetes, although rare, is frequently misdiagnosed as diabetic foot disease. Delays in diagnosis can lead to increased mortality and morbidity. Recognition of suspicious features, such as unusual characteristics of the lesion, longevity despite treatment, and lack of the typical features associated with diabetic foot disease, such as ischaemia and neuropathy, should prompt the clinician to undertake a biopsy to rule out malignancy, including as melanoma.

## Author Contributions

**Ivan R. Bristow:** conceptualization, methodology, investigation, writing – original draft, writing review and editing. **Michelle L. Reynolds:** conceptualization, methodology, investigation, writing – original draft, writing – review and editing. **Matthew Young:** methodology, writing – review and editing.

## Funding

The authors have nothing to report.

## Ethics Statement

The authors have nothing to report.

## Conflicts of Interest

The authors declare no conflicts of interest.

## Data Availability

Data generated in the writing of this paper is available from the authors.
